# Supplementary material for: Comparative Molecular Analysis of Primary Central Nervous System Lymphomas and Matched Vitreoretinal Lymphomas by Vitreous Liquid Biopsy
Source: Int J Mol Sci. 2021 Sep 16;22(18):9992. doi: 10.3390/ijms22189992 (PMC8471952; doi:10.3390/ijms22189992)
Supplement: Supplementary file 1 [file ijms-22-09992-s001.zip › ijms-1344030-supplementary.pdf]

Supplemental Materials

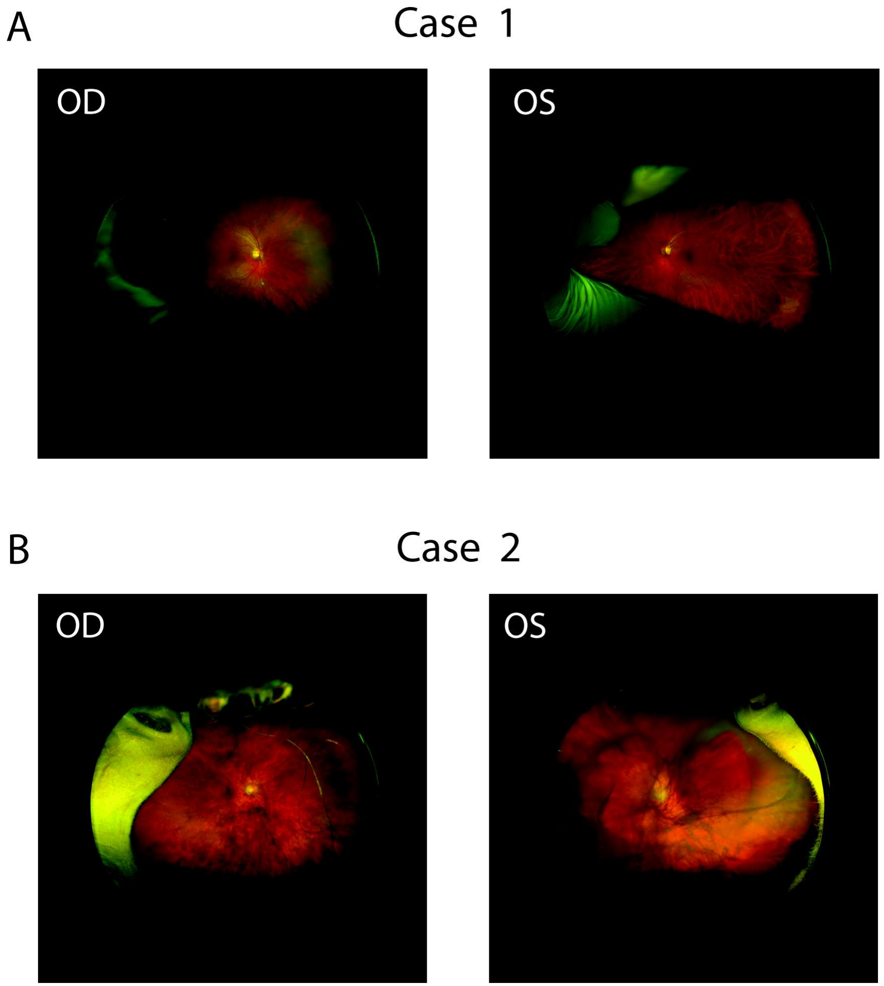

Supplemental Figure S1. Retinal imaging. Widefield retinal imaging for Case 1 (**A**) and Case 2 (**B**) of the right (OD) and left (OS). Case 1 demonstrated scant vitreous cells, which are not visible in the images. Case 2 demonstrated dense vitreous cells and vitreous membranes.

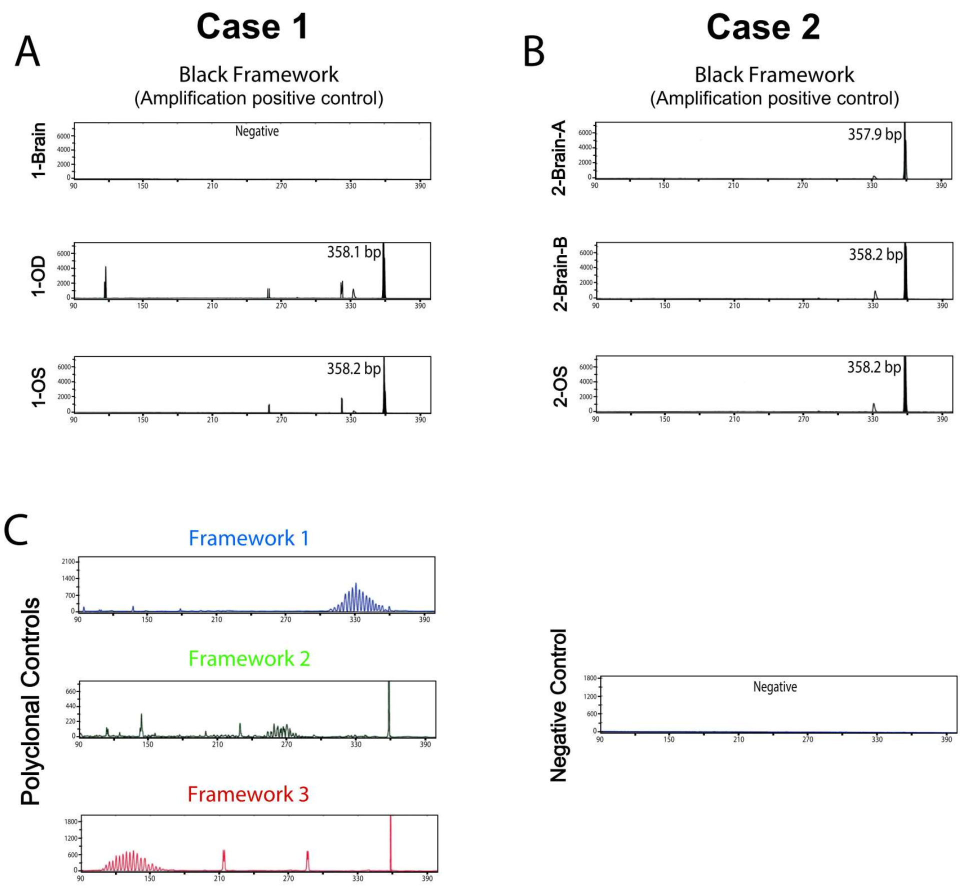

Supplemental Figure S2. Positive control reactions of clonality testing in brain and vitreous liquid biopsy lymphoma samples. DNA obtained from brain PCNSL FFPE tissue and vitreous fluid underwent clonal IGH rearrangement PCR testing. Representative electropherograms for 15% positive control reactions for Case 1 (**A**) and Case 2 (**B**) are shown. Clonal rearrangements were defined as peaks identical between replicates that were at least 2× higher than the third highest peak. The negative controls for each framework (**C**) demonstrate a distribution of multiple clone sizes as would be expected in a specimen with a diverse polyclonal population of B-cells. A no-DNA negative control is shown as well.

Supplemental Table S1. Cytology and Flow cytometry Analysis of Vitreous Liquid Biopsy Samples.

Patient ID	Laterality	Gender	Age	Time Between PCNSL and ocular findings	Cytology Read	Flow Cytometry Read
**Case 1**	OD	Male	53	1 year 7 months	NA	kappa-restricted B cell population, ~48% of total cells
	OS					kappa-restricted B cell population, ~23% of total cells
**Case 2**	OS	Female	78	1 month	Paucicellular	NA

Supplemental Table S2. DNA amount and *IGH* rearrangement PCR testing**.** For each case, brain tissue and vitreous liquid biopsy samples are shown. OS (oculus sinister, left eye), OD (oculus dexter, right eye). *IGH* V(D)J rearrangement amplicon sizes in base pairs (bp) are shown for each framework. *IGH-BCL2* rearrangement status by PCR testing is shown.

Case#	Sample ID	Specimen Type	DNA amount (ng)	Vitreous liq. biopsy Diluted/Undiluted	IGH V(D)J rearrangement	IGH V(D)J rearrangment dominant fragment size (bp)				*IGH-BCL2* t(14:18) oncogenic translocation
						Framework 1	Framework 2	Framework 3	Black framework (+ control)	
1	1-Brain	Brain FFPE Tissue	23.8	N/A	Inadequate Sample	331.1	258.7	117.2 132.8	_	Negative
1	1-OD_Vitreous	Vitreous	31.4	Diluted	Positive	321.7	257.6	116.4	358.1	Negative
1	1-OS_Vitreous	Vitreous	350	Diluted	Positive	321.2	258.3	116.8	358.2	Negative
2	2-Brain_A	Brain FFPE Tissue	610	N/A	Negative	_	_	_	357.9	Negative
2	2-Brain_B	Brain FFPE Tissue	596	N/A	Negative	_	_	_	358.2	Negative
2	2-OS_Vitreous	Vitreous	53.0	First-wash + diluted	Negative	_	_	_	358.1	Negative

Supplemental Table S3. List of genes targeted by the NGS panel. Not all genes have their entire coding sequences targeted. Tumor suppressor genes have nearly 100% coverage of their coding sequences. Other genes are targeted at their hotspot regions or at other regions for copy number assessment.

ABL1	BCL9	CDKN2A	FGFR3	IGF1R	MDM4	NKX2-8	PTEN	STAT3
ACVRL1	BIRC2	CSNK2A1	FLT3	IL6	MED12	NOTCH1	PTPN11	STK11
AKT1	BIRC3	CTCF	FLT3	JAK1	MET	NRAS	RAC1	TERT
ALK	BRAF	CTNNB1	FOXL2	JAK2	MGA	NSD1	RB1	TIAF1
APC	BRCA1	DCUN1D1	GAS6	JAK3	MLL4	PAX5	RET	TP53
APEX1	BRCA2	DNMT3A	GATA2	KIT	MPL	PBRM1	RHEB	U2AF1
AR	C15orf23	EGFR	GATA3	KRAS	MYC	PDCD1LG2	RHOA	VHL
ARHGAP35	CBL	ERBB2	GNA11	KRAS	MYCL1	PDGFRA	RPS6KB1	WT1
ARID1A	CCND1	ERBB2	GNAQ	MAGOH	MYCN	PDGFRA	SETD2	XPO1
ARID1B	CCNE1	ERBB3	GNAS	MAP2K1	MYD88	PIK3CA	SF3B1	ZC3H13
ARID2	CD274	EZH2	HRAS	MAP3K1	MYO18A	PIK3CA	SMO	ZNF217
ATM	CD44	FAT1	IDH1	MAPK1	NCOR1	PIK3R1	SOX2	
ATP11B	CDH1	FBXW7	IDH2	MAX	NF1	PNP	SPEN	
ATRX	CDK4	FGFR1	IFITM1	MCL1	NFE2L2	PPARG	SPOP	
BCL2L1	CDK6	FGFR2	IFITM3	MDM2	NKX2-1	PPP2R1A	SRC	

Supplemental Table S4. NGS of 6 patient tissue and vitreous liquid biopsy samples generated sequencing data with excellent quality parameters. Table shows total reads mapped (to the reference human genome), on-target reads (mapping to the panel target regions), mean depth of sequencing in reads, uniformity (measuring distribution of reads throughout the target regions and defined as percentage of reads having at least 0.2x of the mean coverage), total number of variants detected (low stringency somatic setting), number of DNA bases sequenced, number of DNA bases sequenced with high quality (Q20), number of reads generated, and mean read length in base-pairs (bp).

Patient ID	Sample	Mapped Reads	OnTarget	Mean Depth	Uniformity	Variants	Bases	>=Q20 Bases	Reads	Mean Read Length
**Case 1**	1-OS	1,735,417	99.38%	523.3	89.33%	318	177,493,981	162,699,584	1,740,744	102 bp
	1-OD	2,221,067	99.03%	693.1	95.40%	209	239,868,417	217,004,915	2,227,811	108 bp
	1-Brain	2,535,665	99.24%	783.5	95.16%	225	274,274,414	248,358,012	2,544,890	108 bp
**Case 2**	2-OS	3,134,915	99.28%	985.9	95.56%	224	344,279,316	315,914,417	3,141,690	110 bp
	2-Brain-A	2,998,164	99.26%	924.6	91.36%	227	324,509,886	296,527,900	3,006,388	108 bp
	2-Brain-B	3,021,661	99.33%	925.9	91.60%	232	324,394,630	297,976,844	3,028,482	107 bp
